# *Morc1* as a potential new target gene in mood regulation: when and where to find in the brain

**DOI:** 10.1007/s00221-021-06171-z

**Published:** 2021-07-30

**Authors:** Annakarina Mundorf, Jennifer Koch, Nadja Kubitza, Selina C. Wagner, Michaela Schmidt, Peter Gass, Nadja Freund

**Affiliations:** 1 Division of Experimental and Molecular Psychiatry, Department of Psychiatry, Psychotherapy and Preventive Medicine, LWL University Hospital, Ruhr-University Bochum, Ruhr-Universität Bochum, Experimentelle Und Molekulare Psychiatrie, Universitätsstr. 150, 44801 Bochum, Germany; 2 Institute for Systems Medicine, Department of Medicine, MSH Medical School Hamburg, Hamburg, Germany; 3 Department of Psychiatry and Psychotherapy, Central Institute of Mental Health Mannheim (ZI), Medical Faculty of Mannheim, University of Heidelberg, Mannheim, Germany

**Keywords:** Development, Early life stress, Rat, Depression, Mood regulation

## Abstract

Recent animal and human studies connected the *Morc family CW-type zinc finger 1* (*Morc1*) gene with early life stress and depression. Moreover, the *Morc* superfamily is related to epigenetic regulation in diverse nuclear processes. So far, the *Morc1* gene was mainly studied in spermatogenesis, whereas its distribution and function in the brain are still unknown. In a first attempt to characterize *Morc1* in the brain, we performed a Western Blot analysis as well as a real-time PCR analysis during different stages of development. Additionally, we detected *Morc1* mRNA using real-time PCR in different mood-regulating brain areas in adult rats. We found that MORC1 protein as well as Morc1 mRNA is already expressed in the brain at embryonic day 14 and is stably expressed until adulthood. Furthermore, *Morc1* mRNA is present in many important brain areas of mood regulation like the medial prefrontal cortex, the nucleus accumbens, the hippocampus, the hypothalamus, and the amygdala. The ample distribution in the brain and its molecular structure as a zinc finger protein indicate that *Morc1* might act as a transcription factor. This function and its expression in mood-regulating areas already in the early brain development turn *Morc1* into a possible candidate gene for mediating early life stress and depression.

## Introduction

For a long time, *MORC family CW-type zinc finger 1* (*Morc1*) was only known for its function in mammalian spermatogenesis (Inoue et al. 1999) where its deficiency leads to male-specific germ cell loss and infertility in mice (Pastor et al. 2014). Then, a rat RNA BodyMap scan detected *Morc1* RNA in multiple organs, e.g., the brain, testis, and the highest amount of *Morc1* RNA found in the rat thymus (Yu et al. 2014). Recent psychiatric studies connected *Morc1* to early life stress (ELS) and depression in animals and humans highlighting the potential role of *Morc1* in development and mood regulation (Nieratschker et al. 2014; Schmidt et al. 2016; Mundorf et al. 2018, 2021; Thomas et al. 2020; Bölükbas et al. 2020). However, until now, no study characterized the existence of *Morc1* in the brain.

In general, early childhood is a period of neuroplasticity and maturation of the brain (Johnston 2004). However, multiple factors, one of them being ELS, can cause impairments in neurodevelopment resulting in severe and long-lasting consequences (Teicher 2002). Thus, ELS can induce long-lasting severe physical and mental changes (Carr et al. 2013). Traumatic or uncontrollable stressful events in early childhood, like sexual or physical abuse, loss of a caregiver, or a natural disaster, are associated with the development of depression during adolescence (Putnam 2003; Kendler et al. 2004; Andersen and Teicher 2008). Even neuroanatomical alterations as a result of ELS have been reported resulting mostly in volume reductions of, e.g., the hippocampus, the amygdala, the prefrontal cortex (PFC), and the corpus callosum (Hart and Rubia 2012; Frodl and O’Keane 2013). Neuroanatomical changes subsequently lead to deficits in behavior, memory, and emotional perception (Hart and Rubia 2012). Also, ELS is affecting the activity of certain genes, such as *Morc1*, by altering their DNA methylation pattern. The altered methylation state of specific genes after ELS can subsequently influence the gene expression pattern (Weaver et al. 2004; Labonté et al. 2012; Nieratschker et al. 2014; Mundorf and Freund 2019). Besides changes in gene methylation, the individual genetic background can also play an important role in the etiology of depression and genome-wide association studies have already reported an association between different genetic variation and depression (Power et al. 2017; Ormel et al. 2019).

In a cross-species study, Nieratschker and colleagues found altered methylation of *MORC1* in children`s cord blood and the brains of male rats after exposure to prenatal stress as well as in the blood of male nonhuman primates after ELS (Nieratschker et al. 2014). Moreover, they discovered that certain genetic variants of *MORC1* are connected to major depressive disorder (MDD). In a subsequent study, we were able to validate that a *Morc1* gene knockout leads to depressive-like behavior in adult female mice (Schmidt et al. 2016).

Lower *Morc1* RNA expression in the medial prefrontal cortex (mPFC) was furthermore found after postpartum stress in rats and was accompanied by other subclinical markers connected to depression (Bölükbas et al. 2020).

Studies with humans reinforce the link between *MORC1* methylation and depression as *MORC1* hypermethylation was correlated to signs of depression measured by the Beck Depression Inventory (BDI) in healthy humans (Mundorf et al. 2018). Interestingly, the number of birth complications was also associated with altered *MORC1* methylation (Mundorf et al. 2018). In a multicentric study investigating childhood maltreatment, depressive symptoms, and *MORC1* methylation, no association was found between childhood maltreatment and *MORC1* methylation. However, the association between *MORC1* methylation and depressive symptoms could be confirmed in all three cohorts (Thomas et al. 2020) highlighting the need for further investigation of the role of *Morc1* in ELS and depression. In a follow-up of our previous study (Mundorf et al., 2018), a subsample of the participants was analyzed for neuronal alterations derived from magnetic resonance imaging and neurite orientation dispersion and density imaging in the hippocampus and mPFC (Mundorf et al. 2021). Again, *MORC1* methylation was positively associated with increased BDI scoring, and, interestingly, peripheral *MORC1* methylation was furthermore negatively associated with volume reduction and several neurite orientation dispersion and density markers in the hippocampus and mPFC (Mundorf et al. 2021). Further studies investigating the neuronal implications of *MORC1* in depression are needed to disentangle the potential role of the *MORC1* gene. At this stage, however, even the general distribution of *MORC1* in the brain and during neuronal development is unknown.

The MORC1 protein has been characterized to have a CW-zing finger protein domain (Perry and Zhao 2003). This protein domain is related to chromatin methylation status or early embryonic development (Perry and Zhao 2003), reinforcing the important role of *MORC1* in development. In this study, *Morc1* expression levels in the rat brain from embryonic day 14, postnatal day 2, 22, 42 and >60 were analyzed by Western Blotting and real-time PCR. Moreover, we used real-time PCR to examine the expression pattern of *Morc1* mRNA in different brain regions involved in mood regulation.

## Materials and methods

### Animals

Animals were group-housed on a standard light–dark circle (12/12 h, light period 07:00–19:00) with water and food access ad libitum and constant temperature and humidity conditions (22 ± 2 °C and 55 ± 25%).

As all studies investigating *MORC1* in humans do not report sex differences in *MORC1* methylation (Nieratschker et al. 2014; Mundorf et al. 2018, 2021; Thomas et al. 2020), only male animals were used in this study according to the 3R principles. The only exception is the Morc1 − / − mouse, since male Morc1 − / − mice have a very low life expectancy and are thus very difficult to breed. All experiments were performed according to the principles regarding the care and use of animals adopted by the German Animal Welfare Law for the prevention of cruelty to animals after approval by the LANUV (Landesamt für Natur, Umwelt und Verbraucherschutz Northrhine-Westfalia).

### Experimental design

Western Blotting was performed with protein extracted from whole brain tissue of male Spraque Dawley rats (Charles River Laboratories, Sulzfeld, Germany) at different ages throughout development resulting in the following groups:

- 5 animals from one litter at embryonic day 14 (E14), protein was pooled into one sample
- 4 animals from one litter at postnatal day (P) 2, protein was pooled into one sample
- 1 animal at P22
- 1 animal at P42
- 1 animal at > P60.

For E14 and P2, the brains had to be pooled to enable the extraction of sufficient amounts of protein. No sex determination was conducted for the embryos.

Western blotting was further carried out with the brain of one adult female C57BL/6 N mouse with a Morc1 − / − loss [Morc^Tg(Tyr)1Az/J^, own breeding originally from the Jackson Labs (Bar Harbour, Maine, USA)] to serve as a negative control. As male Morc1 − / − mice have a very low chance of survival, females had to be analyzed. Protein extract from testes of an adult (> P60) male rat served as a positive control.

For real-time (rt) PCR analysis, protein extracted from male Spraque Dawley rat brains (Charles River Laboratories, Sulzfeld, Germany) was analyzed. For the analysis of *Morc1* mRNA expression throughout life (rtPCR part I, Fig. 2A), mPFC RNA was extracted from three animals per age resulting in the following groups:

- 3 animals at E14 (cortices)
- 3 animals at P2
- 3 animals at P22
- 3 animals at P42
- 3 animals at P62.

For the rtPCR analysis of *Morc1* expression levels of different brain regions (rtPCR part II, Fig. 2B), the brains of three male adult rats (> postnatal day 60) were dissected and included for analysis. To avoid genetic influences, the three rats per group were not related. Furthermore, rats were housed in different groups with their respective siblings. Again, no sex determination was conducted for the embryos.

### Western blotting

Protein was extracted using the whole brain including the cerebellum. To obtain sufficient protein for all ages, the protein of five E14 embryo brains from one litter were pooled. Furthermore, the protein was pooled for the P2 sample from four male pups from one litter. For the other ages, as well as the Morc1 (−/−), extracted protein was gained from one animal in sufficient quantities. All samples were homogenized, and protein was extracted with a Protease Inhibitor (Protease Inhibitor Cocktail Tablets complete from Roche, 1 tablet diluted in 2 ml distilled H_2_0) and radioimmunoprecipitation assay buffer. An 8% SDS-polyacrylamide gel was produced (ingredients from Roth). As marker Color Presteined Protein Standard (P7712S, New England Biolabs) was used (245–11 kDa). Previous experiments revealed that for brain samples, 100 µg of protein was necessary for a successful anti-MORC1 staining, whereas 20 µg protein of testis was sufficient. All samples were loaded with protein mixture and 5 µl Lämmli Buffer on an 8% SDS-polyacrylamide gel first at 100 V for 20 min, and then for 1 ^1^/_2_ h at 120 V (BIO-RAD Power Supply). The protein was transferred from the gel to a methanol-activated PVDF- Membrane (0.45 µm, Carl Roth GmbH) for 1 h at 100 V on ice. Afterward, the membrane was blocked for 1 h with gentle agitation in 5% non-fatty milk in 1 × TBST at room temperature. Then, the membrane was incubated for one night at 4 °C with the primary antibody anti-MORC1 gained out of rabbit (1:500; 14,080–1-AP, Proteintech). On the next day, after washing, the secondary antibody anti-rabbit HPR from goat (1:5000, 65–6120, Invitrogen) was incubated in 2% non-fatty milk in 1 × TBST for 1 h. After stripping, blots were incubated overnight with anti-beta-Actin gained from rabbit (1:1500, 4967S, Cell Signaling) with gentle agitation in 0.5% BSA in 1 × TBST. The second antibody was incubated in 0.5% BSA in 1 × TBST for 1 h. For chemiluminescent detection, the membrane was incubated with the enhanced chemiluminescent Thermo Scientific™ SuperSignal™ West Pico PLUS kit (1:1, 34,087, Thermo Fisher). Blot imaging was then performed using the ChemiDoc™ MP Imaging System (BIO-RAD).

### Real-time PCR

For rtPCR quantification of *Morc1,* RNA brain regions were dissected from three adult (> P60) male rats. The mPFC, nucleus accumbens (NAc), hippocampus, amygdala, and hypothalamus were dissected according to the rat brain atlas (Paxinos and Watson 2006). Additionally, the mPFC of three male rats of each developmental stage (E14, P2, P22, P42, and P62) were dissected. RNA isolation was performed using the NucleoSpin® TriPrep Kit (Macherey–Nagel, Düren, Germany) with slight modifications, meaning that 40 μl of RNase-free water was added to obtain RNA. After the quantification and quality assessment, RNA was reverse transcribed to cDNA using the High-Capacity RNA-to-cDNA™ Kit (Thermofisher Scientific, Darmstadt, Germany) according to the manufacturer’s protocol. Experiments for establishing *Morc1* rtPCR revealed a minimum of 60 ng RNA needed per sample to detect stable results (see also Bölükbas et al. 2020). Thus, 60 ng cDNA was used to measure *Morc1* mRNA levels in all regions and ages for all animals separately. For rtPCR analysis, TaqMan™ Gene Expression Master Mix (Thermofisher) and TaqMan gene expression assay for *Morc1* (Rn01474745_m1) as well as for *Glyceraldehyde-3-phosphate dehydrogenase* (Rn01775763_g1) and *Actin, beta* (Rn00667869_m1) serving as housekeeping genes were used. The CFX Connect Real-Time PCR System (Bio-Rad) was used according to the manufacturer’s protocol. All samples and gene assays were assayed in duplicates. RNA levels were quantified by the number of cycle thresholds (Delta CT method). The ∆Ct value of each sample was calculated by subtracting the mean Ct value of the housekeeping genes from the mean Ct value of *Morc1.* To account for individual plate differences, the three P62 mPFC samples were run on all plates and values were calculated as percent expression related to the mean expression of P62 mPFC.

## Results

### Western blotting

**Fig. 1:**
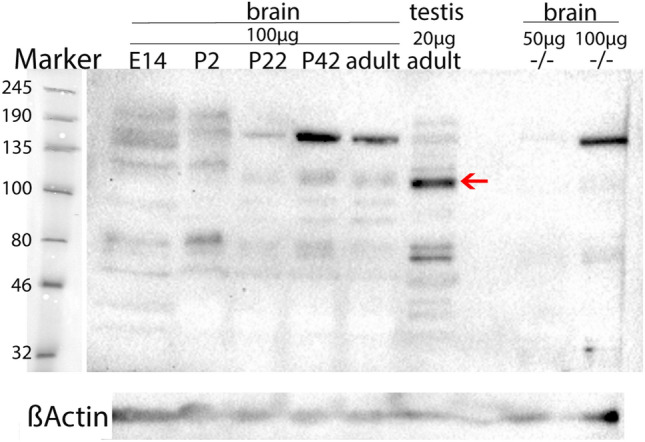
Western blot imaging of MORC1 protein [anti-MORC1 from rabbit (1:500; 14,080–1-AP, Proteintech)] in rat brain at the age of E14 (pooled from 5 embryos), P2 (pooled from 4 pups), P22, P42, adult (P > 60), and protein from an adult rat testis (1 animal each). Moreover, MORC1 protein was stained in one adult female C57BL/6 N Morc1 (−/−) brain using 50 µg and 100 µg protein. Imaging was performed with the ChemiDoc™ MP Imaging System (BIO-RAD). A prominent band was detected at 110 kDa (red arrow) in all rat samples but not the Morc1 (−/−) sample. Beta-actin staining revealed prominent bands at 45 kDa (ßActin) in all samples

The rat brain samples revealed a prominent band at 110 kDa at all age stages (E14, P2, P22, P42, and P > 60) (Fig. 1, red arrow). Beta-actin staining revealed prominent bands in all samples at 45 kDa confirming an equal protein amount per sample (Fig. 1). Interestingly, a light band at 110 kDa was also detected in the Morc1 (−/−) mouse brain sample when the total protein amount was increased to 100 µg (Fig. 1). The strongest band was seen in the rat testis sample using 20 µg of the total protein. All brain protein samples also show a prominent band at around 180 kDa which is especially prominent in P42 and adult brain samples when applying 100 µg of total protein. This band is not detected when applying 50 µg from the Morc1 (−/−) protein sample. However, multiple bands besides the one at 110 kDa were detected, possibly due to the polyclonal properties of the chosen antibody and the high amount of lysate loaded for the brain protein samples.

### Real-time PCR

**Fig. 2:**
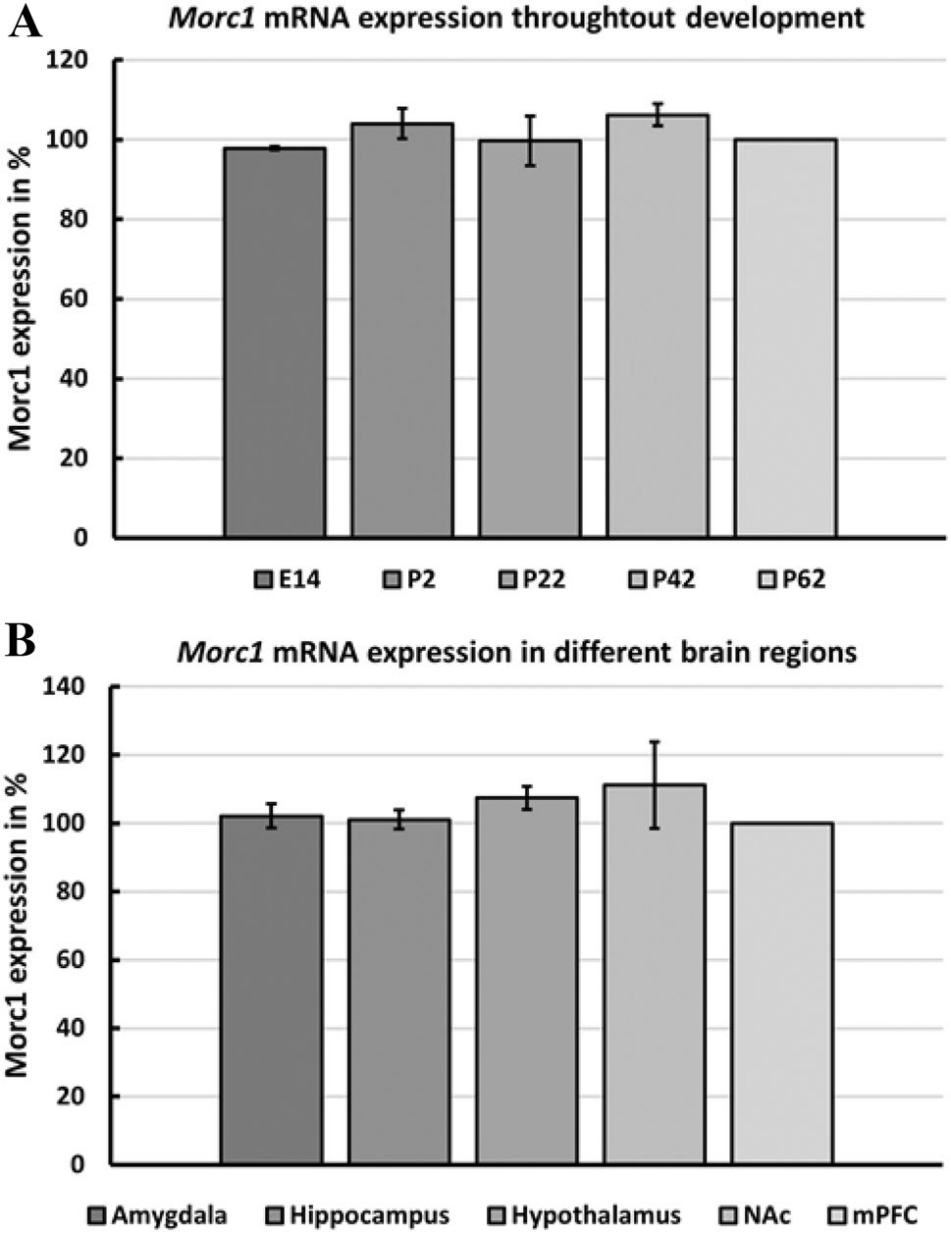
Relative *Morc1* mRNA expression in the rat brain. Values are given as percent expression related to the mean expression of P62 mPFC. The mean value of the three investigated animals per age/region is given as *Morc1* expression in % as well as the standard deviation (SD). **A** *Morc1* expression in different developmental stages. E: embryonic stage, P: postnatal day. **B** *Morc1* expression in different brain regions in adult male rats. *NAc* nucleus accumbens, *mPFC* medial prefrontal cortex

*Morc1* mRNA was detected in all developmental stages and all brain regions using rtPCR. It seems that *Morc1* is expressed almost equally in all developmental stages (Fig. 2A). However, slightly more *Morc1* mRNA was detected in the developmental stages P2 and P42 compared to other ages investigated. The *Morc1* expression levels seem to be also very equally in all brain regions (Fig. 2B).

## Discussion

Western blotting of the mouse and rat tissue detected prominent bands at 110 kDa which correspond to the estimated molecular weight of *Rattus norvegicus* MORC1 protein of 109 kDa (NCBI 2016). Less protein was needed for detecting MORC1 in rat testis than for brain samples as the testis is known to contain a high amount of *Morc1* RNA (Yu et al. 2014). This finding is in line with the high amount (60 ng) of RNA needed for rtPCR analysis of *Morc1* in the brain.

However, finding a promising antibody for anti-MORC1 staining proved to be difficult. As the used antibody is polyclonal thus targeting multiple epitopes within the same antigen, multiple bands besides the one at 110 kDa were detected. Moreover, the high amount of brain lysate needed to detect MORC1 protein can lead to increased non-specific binding of the antibody, resulting in multiple bands as detected in our Western Blotting. This hypothesis is in line with the fact that the prominent band at 180 kDa is less prominent when loading a lower amount of lysate (20 µg from the testis and 50 µg Morc1 -/- brain), indicating that the high amount of 100 µg lysate results in non-specific staining besides the band at 110 kDa. Thus, investigating MORC1 protein expression in different brain regions, even though it is very important to analyze, would not have been accurate given the problematic balance between the high quantity needed and the increasing non-specific bindings.

Interestingly, Western blotting against the MORC1 protein displayed a slight band at 110 kDa in the Morc1 (−/−) mouse, too, when increasing the amount of total protein to 100 µg. Given the properties of the Morc1 (−/−) knockout construct, this might not be surprising. For the knockout of MORC1, the Exons 2–4 were deleted and replaced by a sequence of almost the same length in the 3` region of MORC1 cDNA which leads to an aberrant mRNA transcript (Inoue et al. 1999). Thereby, the Morc1 (−/−) mouse may express a shortened protein which in turn is not functional as it would be lacking the first residues of the MORC N-terminal region essential for functioning (Inoue et al. 1999). However, Pastor and colleagues (2014) did not detect MORC1 protein in the Morc1 (−/−) mouse testis using immunohistochemistry (Pastor et al. 2014). Yet, the antibody used by Pastor et al. was self-constructed and was thus not available for replication. Our commercially available antibody might bind to different sites of the protein and therefore also detect the truncated, unfunctional protein of the knockout. Moreover, Western blotting might be more sensitive than immunohistochemistry as much more total protein was used for detection in our study.

Detecting *Morc1* RNA in the amygdala, and the hippocampus suggests a role of *Morc1* in mood regulation as these regions are part of the limbic system (Borsook et al. 2016). Interestingly, parts of the mesolimbic dopaminergic pathway, such as the NAc, the amygdala, and the hypothalamus, also express *Morc1* RNA. These structures are part of the reward circuitry whose function is impaired in MDD (Dichter et al. 2012).

In general, ELS can lead to altered methylation state of genes (Weaver et al. 2004; Labonté et al. 2012; Nieratschker et al. 2014), one of them being the *MORC1* gene (Nieratschker et al. 2014; Mundorf et al. 2018), and thereby affect gene expression. So far, the absence of MORC1 protein leads to depressive-like behavior (Schmidt et al. 2016), and certain genetic variants of *MORC1* are connected to MDD (Nieratschker et al. 2014). Recently, we could report an association between the hypermethylation of *MORC1* and higher BDI scores, as well as changes in the methylation pattern after ELS (Mundorf et al. 2018). All these findings reinforce the important role the *MORC1* gene plays during development and in the development of depression.

Based on the performed Western blotting including different developmental stages of the brain, it was possible to detect MORC1 protein expression at all different ages. The time points investigated by Western Blotting were chosen to cover the most important neurodevelopmental stages as prenatal (E14), postnatal (P2), early childhood (P22, after weaning), early adolescence (P42), and early adulthood (> P60). Given the fact that MORC1 is expressed in early embryonic development, as well as in the entire brain, the gene may take an important part in early neurodevelopment. As the expression does not seem to differ during development, altered MORC1 expression induced by experienced ELS could be triggering structural changes. Impaired signal processing in specific brain regions could then lead to severe dysfunctions and structural changes, such as the reduced volume of the hippocampus, amygdala, PFC, and corpus callosum reported after ELS (Hart and Rubia 2012; Frodl and O’Keane 2013). It is already well known that dysfunction of transcriptional genes leads to impaired plasticity and thereby to severe disorders (Johnston 2004) and cognitive impairment (Johnston et al. 2003). Given these facts, it does not seem too far-fetched to assume that *MORC1* as a transcription factor might be involved in the development of severe disorders like MDD.

Generally, there are already several studies linking ELS with depressive-like behavior and dysfunctional brain networks, but the pathogenesis is still unclear. A study in rat pups discovered that ELS results in a dysfunction of prefrontal and mesolimbic regions in the young rat brain, which may contribute to behavioral changes later in life (Spivey et al. 2011). The mesolimbic system is one region where *Morc1* was found in our study, so altered *Morc1* expression after ELS in mesolimbic structures could be one reason for behavioral changes. Furthermore, in male rats, ELS disrupts the dendritic morphology of neurons in the PFC, hippocampus, and NAc (Monroy et al. 2010). A disrupted function in any of these regions leads to MDD symptoms. Altered expression of *Morc1*, specifically given its role as a transcription factor, could be the reason for this dysmorphology.

Taken together, *Morc1* seems to be present in many important areas whose dysfunction can lead to the clinical picture of MDD. The wide distribution in the brain and its molecular structure indicate that *Morc1* acts as a transcription factor in the brain, as well. Therewith, *Morc1* would be able to influence gene expression patterns within pathways involved in mood regulation and emotion and might be the decisive structure that causes MDD after exposure to ELS. This hypothesis is in line with our reported negative association between *MORC1* methylation and macro- and micro-structural markers in subclinical participants (Mundorf et al. 2021). As high values of total protein are needed for the detection of *Morc1* expression, no region-specific protein expression pattern could be analyzed during development. Moreover, only regions providing at least 60 ng of total RNA were included for rtPCR analysis. Thus, more studies investigating different brain regions are needed to support *Morc1*s role in the brain.

Given that MORC1 protein already exists in early embryonic stages, ELS might influence its expression pattern right from the beginning and thereby impair regular brain development. Besides recent human studies investigating *MORC1* which significantly increased its connection to mood disorders, its functional role in the brain is still unknown. Therefore, more studies investigating the *Morc1* gene in the brain are highly needed.

## Data Availability

Data are available upon request from the corresponding author.

## Abbreviations

ELS: Early life stress
PFC: Prefrontal cortex
MDD: Major Depressive Disorder
mPFC: Medial prefrontal cortex
MORC1: MORC family CW-type zinc finger 1
NAc: Nucleus accumbens
RT: Real time
